# Can augmented renal clearance be detected using estimators of glomerular filtration rate?

**DOI:** 10.1186/s13054-020-03057-4

**Published:** 2020-06-18

**Authors:** Matthias Gijsen, Alexander Wilmer, Geert Meyfroidt, Joost Wauters, Isabel Spriet

**Affiliations:** 1 Pharmacy Department, Department of Pharmaceutical and Pharmacological Sciences, University Hospitals Leuven and Clinical Pharmacology and Pharmacotherapy, KU Leuven, Leuven, Belgium; 2 Clinical Division and Laboratory of Intensive Care Medicine, University Hospitals Leuven and Academic Department of Cellular and Molecular Medicine, KU Leuven, Leuven, Belgium

**Keywords:** Augmented renal clearance, Intensive care unit, Creatinine clearance, Glomerular filtration rate

Estimators of glomerular filtration rate (GFR) have been shown to be flawed in critically ill patients, especially for augmented renal clearance (ARC), commonly defined as a measured urinary creatinine clearance (CrCl) ≥ 130 ml/min/1.73 m^2^ [1]. Therefore, measuring CrCl should be performed in daily practice on the intensive care unit (ICU). However, many ICUs still rely on estimating formulae to monitor GFR [1, 2]. As estimators underestimate measured CrCl in ARC patients, ARC might remain unrecognized and lead to subtherapeutic plasma levels of drugs with predominant renal clearance [3]. Therefore, the aim of this study is to define the most precise GFR estimator, which can then be used to detect ARC when measured CrCl is unavailable.

We performed a multicenter retrospective registry-based [4] cohort study in adult ICUs from 3 tertiary university-affiliated hospitals in Belgium (Leuven, Ghent, Antwerp). All consecutive patients admitted between January 2013 and December 2015 were screened for eligibility. All patients ≥ 18 years old and having at least one measured 24-h urinary CrCl (CrCl_24h_) were included. Agreement between CrCl_24h_ and formulae estimating renal function, i.e., Cockcroft–Gault (CG), Chronic Kidney Disease Epidemiology Collaboration (CKD-EPI), and Modification of Diet in Renal Disease Study (MDRD), was evaluated on all included ICU days. For the estimator with the best precision, a cut-off for ARC with optimal specificity and sensitivity was identified, by calculating the Youden index [5]. Predictions for ARC using the cut-off value were compared to the actual presence of ARC based on the CrCl_24h_. Cut-off values with either very high sensitivity (> 95%) or specificity (> 95%) were also identified. Finally, the performance of these cut-offs was evaluated in an external single-center (Leuven, January 2016–December 2016) validation set by receiver-operating characteristics (ROC) curve analysis, using 2000 bootstrap replicates. The same inclusion and exclusion criteria as described above were applied.

A total of 51,604 ICU days were included to define a cut-off. Agreement analysis between CrCl_24h_, the clinical reference, and the formulae estimating renal function is shown in Table 1. None of the estimators were precise (i.e., standard deviation of the mean bias was large for all estimators), with the CKD-EPI formula performing best over the whole CrCl_24h_ range, and for ARC specifically, as illustrated in Fig. 1a. Hence, the CKD-EPI formula was selected for further analysis. In the validation set, 10,503 ICU days were included. For the CKD-EPI formula, the optimal cut-off for ARC was 96.5 ml/min/1.73 m^2^. This cut-off showed a sensitivity of 86.6% [85;88.1] and a specificity of 71% [70;71.9]. The cut-off values with very high sensitivity and specificity were 87.3 ml/min/1.73 m^2^ (sens, 95.8% [95;96.7]; spec, 57.6% [56.6;58.7]) and 125.2 ml/min/1.73 m^2^ (sens, 31.4% [29.4;33.5]; spec, 95.2% [94.7;95.6]), respectively. The ROC curve analysis including the cut-off values is shown in Fig. 1b. Evaluating the optimal cut-off in the validation set, we found that the proportion of accurate predictions for ARC decreased during the first 2 weeks of ICU stay. The is due to an increased false positive rate (Day-1, 16%; Day-14, 49%).

**Table 1:** Agreement analysis between CrCl_24h_ and formulae estimating renal function

	All ICU days (*n* = 51,604)	CrCl_24h_ < 130 ml/min/1.73 m^2^ (*n* = 41,290)	CrCl_24h_ ≥ 130 ml/min/1.73 m^2^ (*n* = 10,314)
Median (IQR) (ml/min/1.73 m^2^)			
CrCl24h	73 (37;118)	58 (30;88)	166 (145;200)
CrCl_CG_	83 (50;127)	70 (43;103)	145 (116;183)
eGFR_MDRD_	87 (50;130)	72 (42;109)	143 (115;185)
eGFR_CKD-EPI_	88 (51;108)	75 (43;99)	116 (104;130)
Correlation with CrCl_24h_ = Spearman correlation coefficient			
CrCl_CG_	0.63°	0.62°	0.18°
eGFR_MDRD,_	0.59°	0.60°	0.15°
eGFR_CKD-EPI_	0.69°	0.72°	0.19°
Mean bias (95% CI) = mean difference CrCl_24h_ – estimator (ml/min/1.73 m^2^)			
CrCl_CG_	− 11 (− 11;-10)	− 20 (− 20;-19)	25 (23;27)
eGFR_MDRD_	− 14 (− 15;− 14)	− 23 (− 23;− 23)	21 (19;23)
eGFR_CKD-EPI_	3 (3;4)	− 12 (− 13;− 12)	66 (64;67)
Precision = SD of the bias (ml/min/1.73 m^2^)			
CrCl_CG_	55	41	83
eGFR_MDRD_	61	46	94
eGFR_CKD-EPI_	48*	26*	62*
Accuracy = percentage within 30% of CrCl_24h_			
CrCl_CG_	47	45	58
eGFR_MDRD_	45	43	56
eGFR_CKD-EPI_	50	51	45

*n* number of ICU days; *CrCl*_*24h*_ creatinine clearance measured by 24-h urine collection, corrected for body surface area; *IQR* interquartile range; *CrCl*_*CG*_ estimated creatinine clearance by the Cockcroft – Gault formula, corrected for body surface area; *eGFR*_*MDRD*_ estimated glomerular filtration rate by the 4-variable Modification of Diet in Renal Disease formula; *eGFR*_*CKD-EPI*_ estimated glomerular filtration rate by the Chronic Kidney Disease Epidemiology Collaboration formula; *SD* standard deviation; *CI* confidence interval

°*p* < 0,001

*Best performing

**Fig. 1:**
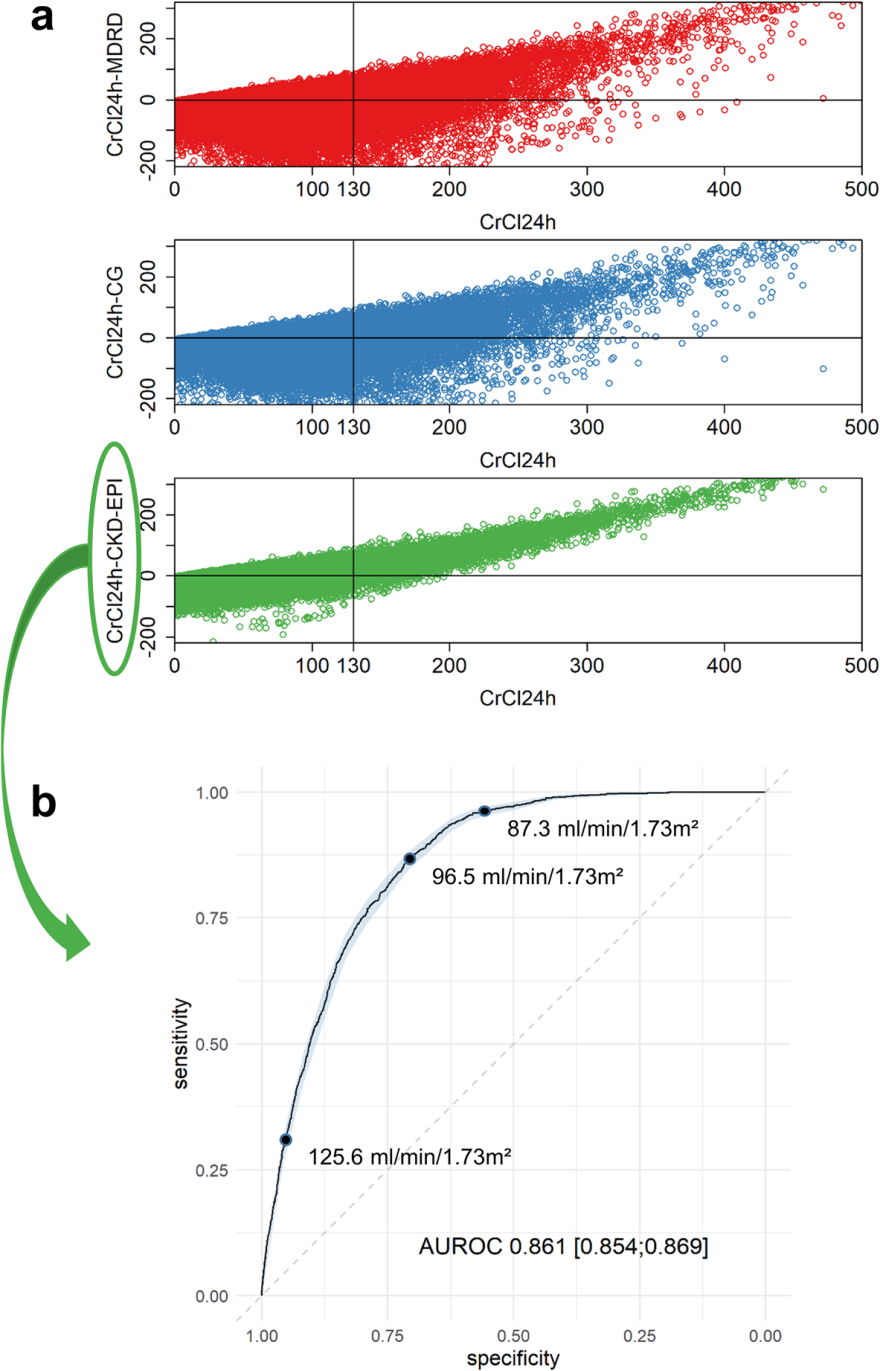
**a** Bias in function of CrCl_24h_ for the three formulae estimating renal function. Top: MDRD (ml/min/1.73 m^2^); mid: CG corrected for a body surface area of 1.73 m^2^ (ml/min/1.73 m^2^); bottom: CKD-EPI (ml/min/1.73 m^2^). **b** Receiver operating characteristics curve analysis for the CKD-EPI formula. The shaded area represents the 95% confidence intervals. The dots represent the cut-off values for optimized sensitivity and specificity, very high (> 95%) sensitivity and very high (> 95%) specificity

Overall, there was poor agreement between CrCl_24h_ and GFR estimators, confirming previous literature [1]. However, the CKD-EPI formula, which is the “least worse” alternative to CrCl_24h_, provided a cut-off with reasonable performance to detect ARC. Depending on the clinical context, this cut-off can be adapted to increase sensitivity or specificity. When applying this cut-off, the user should note that the accuracy decreases over time during the first 2 weeks of ICU stay. Hence, its largest benefit lies in the beginning of ICU stay. The presented CKP-EPI cut-off can be used to guide upfront increased antimicrobial dosing in patients presenting with ARC early upon ICU admission, when CrCl_24h_ is not available.

## Data Availability

The datasets used and/or analyzed during the current study are available from the corresponding author upon reasonable request.

## Abbreviations

GFR: Glomerular filtration rate
ARC: Augmented renal clearance
CrCl: Creatinine clearance
ICU: Intensive care unit
CrCl_24h_: Measured 24-h urinary creatinine clearance
CG: Cockcroft–Gault formula
CKD-EPI: Chronic Kidney Disease Epidemiology Collaboration formula
MDRD: Modification of Diet in Renal Disease Study formula

## References

[1] Bilbao-Meseguer I, Rodriguez-Gascon A, Barrasa H, Isla A, Solinis MA (2018). Augmented renal clearance in critically ill patients: a systematic review. Clin Pharmacokinet.

[2] Ruiz S, Minville V, Asehnoune K, Virtos M, Georges B, Fourcade O (2015). Screening of patients with augmented renal clearance in ICU: taking into account the CKD-EPI equation, the age, and the cause of admission. Ann Intensive Care.

[3] Udy AA, Roberts JA, Lipman J (2013). Clinical implications of antibiotic pharmacokinetic principles in the critically ill. Intensive Care Med.

[4] M@tric project [Available from: https://www.matric.be/]. Accessed 4 Nov 2019.

[5] Fluss R, Faraggi D, Reiser B (2005). Estimation of the Youden index and its associated cutoff point. Biom J.

